# Hydration differences between the major and minor grooves of DNA revealed from heat capacity measurements

**DOI:** 10.1007/s00249-018-1340-0

**Published:** 2018-12-14

**Authors:** Anatoliy I. Dragan, Christopher M. Read, Colyn Crane-Robinson

**Affiliations:** 1 Institute of High Technologies, Taras Shevchenko National University of Kyiv, 64, Volodymyrs’ka St., Kyiv, 01601 Ukraine; 2 Institute of Molecular Biology and Genetics, NASU, 150, Zabolotnogo Str., Kyiv, 03680 Ukraine; 3 Biophysics Laboratories, School of Biology, University of Portsmouth, Portsmouth, PO1 2DT UK

**Keywords:** Heat capacity, Hydration, Proteins, DNA

## Abstract

The nature of water on the surface of a macromolecule is reflected in the temperature dependence of the heat effect, i.e., the heat capacity change, Δ*C*p, that accompanies its removal on forming a complex. The relationship between Δ*C*p and the nature of the surface dehydrated cannot be modeled for DNA by the use of small molecules, as previously done for proteins, since the contiguous surfaces of the grooves cannot be treated as the sum of small component molecules such as nucleotides. An alternative approach is used here in which Δ*C*p is measured for the formation of several protein/DNA complexes and the calculated contribution from protein dehydration subtracted to yield the heat capacity change attributable to dehydration of the DNA. The polar and apolar surface areas of the DNA dehydrated on complex formation were calculated from the known structures of the complexes, allowing heat capacity coefficients to be derived representing dehydration of unit surface area of polar and apolar surface in both grooves. Dehydration of apolar surfaces in both grooves is essentially identical and accompanied by a reduction in Δ*C*p by about 3 J K^−1^ mol^−1^ (Å^2^)^−1^, a value of somewhat greater magnitude than observed for proteins {Δ*C*p = − 1.79 J K^−1^ mol^−1^ (Å^2^)^−1^}. In contrast, dehydration of polar surfaces is very different in the two grooves: in the minor groove Δ*C*p increases by 2.7 J K^−1^ mol^−1^ (Å^2^)^−1^, but in the major groove, although Δ*C*p is also positive, it is low in value: + 0.4 J K^−1^ mol^−1^ (Å^2^)^−1^. Physical explanations for the magnitudes of Δ*C*p are discussed.

## Introduction

Protein folding is typically characterized by a significant reduction in the heat capacity of the system. Negative values of Δ*C*p, i.e., a reduction in the heat of folding with increase in the temperature Δ*C*p = δΔ*H*/δ*T*, are not principally due to additional conformational restraints to the polypeptide chain on folding, but to changes in the state of hydration (Makhatadze and Privalov 1995). Dominantly, this is loss of water from hydrophobic groups as they come together inside the folded core of the protein and a heat capacity decrement is regarded as a defining feature of protein folding. However, analysis of polar interactions in proteins led to the conclusion that their formation is associated with a heat capacity increase—but since the positive values of Δ*C*p for polar interactions are not as large as the negative values characterizing apolar interactions (per Å^2^ of interacting protein surface) the latter dominate the heat capacity change on folding (Spolar et al. 1992; Murphy and Friere 1992; Privalov and Makhatadze 1992; Makhatadze and Privalov 1995). Equations have been established relating the changes in apolar and polar water accessible surface areas (ΔASA) to the resulting change in the heat capacity as a protein folds and are effective predictors of experimental Δ*C*p values. These observations can be summarized by saying that changes in the heat capacity of proteins and their complexes reflect alterations in their state of hydration, i.e., heat capacity changes are a proxy for changes in hydration.

When proteins associate (non-covalently) the circumstances are closely akin to the folding of individual polypeptide chains and the equations applicable to the folding of individual chains are equally effective predictors of the associated changes in the heat capacity accompanying complex formation. The formation of protein/DNA complexes is also associated with significant reductions in the heat capacity, likewise in consequence of the dehydration of hydrophobic surfaces (Ha et al. 1989; Spolar and Record 1994; Ladbury et al. 1994). However, the equations established for proteins and protein/protein interactions are not good predictors of the heat capacity changes observed for the binding of protein domains (DBDs) to duplex DNA (Ladbury et al. 1994; Morton and Ladbury 1996; O’Brien et al. 1998; Bergqvist et al. 2004) and this discrepancy has been assigned to the presence of residual waters at the protein/DNA interface and in peripheral polar environments (Morton and Ladbury 1996; Bergqvist et al. 2004).

A priori, it seems unlikely that heat capacity coefficients derived from the small compounds used to model proteins would be appropriate to the surface of the DNA grooves (Prabhu and Sharp 2005). Determination of such coefficients using small compounds modeling the components of DNA (bases, sugars) is unlikely to be helpful: the grooves are deep cavities with regular and closely spaced groups, so cooperativity in the binding of surface water is expected to occur and the additivity assumption would break down. A quite different approach is, therefore, required to determine the precise characteristics of dehydrating the surface of DNA in terms of the surface areas that become dehydrated in the major and the minor grooves. This article makes use of existing heat capacity data to generate relationships between the loss of accessible apolar and polar surface area in both the major and the minor grooves of DNA and the resulting contribution to the magnitude of the heat capacity change that occurs on complex formation with DNA binding domains (DBDs). It, therefore, represents a study of hydration in the major and minor grooves.

For protein folding, the total heat capacity effect, Δ*C*p(*T*), is formalized in equations of the type:

1 $$ \Delta C{\text{p}}\left( T \right) = \varSigma \Delta \left( {\text{ASA}} \right)^{i} \times C{\text{p}}^{i} \left( T \right), $$

where the coefficient *C*p^*i*^(*T*) represents the heat capacity effect per Å^2^ of surface of defined type i and Δ(ASA)^*i*^ is the change (reduction) in the accessible surface area of that type that becomes buried. Three types of surface have been recognized as distinct: aliphatic (non-polar), aromatic, and polar/charged, with the result that such a predictive equation has three terms. However, for many purposes aromatic surfaces can be regarded as non-polar, reducing the equations to two terms. For application to folding a protein of known structure, computer programs are used that roll a 1.4 Å sphere (representing a water molecule) over the surface of the unfolded and folded chains and the difference in their water accessible surface areas, Δ(ASA)^*I*^, in consequence of folding thereby evaluated for both categories of surface.

To interpret experimental Δ*C*p values accompanying formation of DBD–DNA complexes—the structures of which are known from X-ray and/or NMR studies—in terms of changes in accessible surface area requires that both interacting components be fully folded. However, significant refolding of DBDs frequently occurs on forming DNA complexes, (Spolar and Record 1994; Privalov et al. 1999; Privalov and Crane-Robinson 2018), making a considerable contribution to the observed heats of binding observed in the titration calorimeter, with the result that the temperature dependence does not reflect the interaction of fully folded components. It is critical, therefore, to select for analysis only complexes for which correction for refolding has been applied by subtracting refolding heats—separately measured in the scanning calorimeter—from ITC derived heats of association. In a few of the cases selected, care was taken by the authors to ensure that the unbound protein is already in a fully folded state, e.g., by restricting enthalpy measurements to low temperatures. If the DBD is a short peptide, the refolding issue is absent. In both circumstances the temperature dependence of the binding enthalpies, i.e., Δ*C*p values—represent the binding of fully folded protein to DNA. Only complexes fulfilling these criteria were accepted into the analysis.

## Methodology

### Relating measured heat capacity changes for protein/DNA complexes to the dehydration of non-polar and polar surface of the two DNA grooves

The magnitude and sign of the *C*p^*i*^ (*T*) coefficients (Eq. 1) for protein folding have been assessed in three separate studies: Murphy and Friere (1992), Spolar et al. (1992) and Makhatadze and Privalov (1995). The averaged values of the above three studies can be represented by the equation:

2 $$ \Delta C{\text{p}}^{\text{prot}} \left( { 2 5\;^\circ {\text{C}}} \right){ = } - 1. 7 9\pm 0. 4 0\cdot \Delta {\text{ASA}}_{\text{apolar}} { + 0} . 9 8\pm 0. 3 5\cdot \Delta {\text{ASA}}_{\text{polar}} , $$

in which the *C*p^*i*^ (*T*) coefficients are expressed in J K^−1^ mol^−1^ (Å^2^)^−1^ and ΔASA are in Å^2^.

This equation was used to obtain the contribution from dehydration of the proteins in the selected complexes and the values obtained then subtracted from the total (observed) heat capacity change, Δ*C*p^obs^, to yield the difference (Δ*C*p^DNA^) that represents the heat capacity change resulting from dehydration of the DNA surface in forming the complex. To deconvolute values of Δ*C*p^DNA^ into contributions from apolar and polar surface dehydration, i.e., to develop equations similar to (2) but for DNA, reductions in the accessible surface areas (ΔASA) of the DNA components of the complexes were calculated for the apolar and polar surface of the minor and major grooves of the individual complexes. Combining the data for the several major groove complexes (six in number) and, separately, the several minor groove complexes (five in number) then allowed evaluation of the *C*p^*i*^ (*T*) coefficients for apolar and polar surface in the two DNA grooves.

## Results

**Fig. 1:**
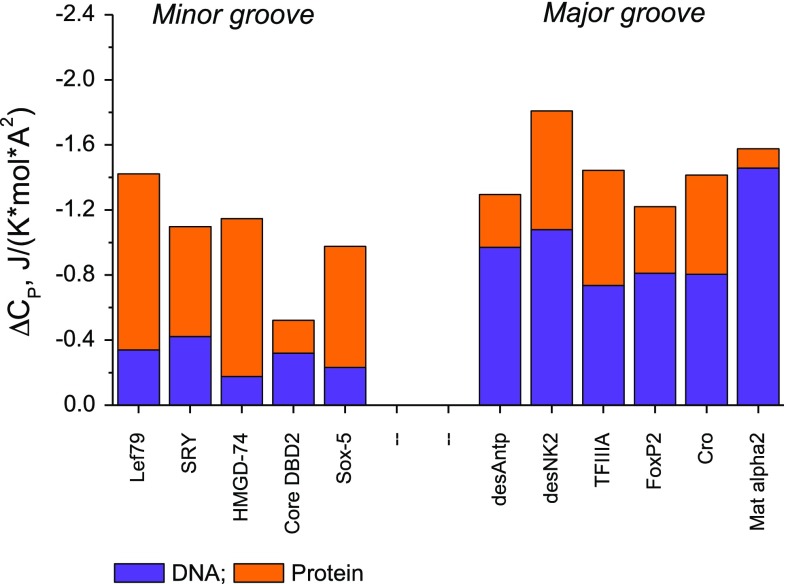
Total surface-normalized (i.e., per A^2^) of observed heat capacity changes, Δ*C*p^obs^, for binding DBDs to their optimal recognition target sequences. The contribution from the protein components (orange), Δ*C*p^prot^, was calculated from the averaged protein Eq. (2). The DNA contributions, Δ*C*p^DNA^, (blue) were obtained by subtraction from Δ*C*p^obs^. The interfacial areas used for normalization were averages of the protein and DNA contact areas. For details see Table 1

Data for experimental heat capacity changes, Δ*C*p^obs^, were normalized to unit Å^2^ of interface to facilitate comparison between complexes of varying size and are summarized in Fig. 1, separated into major and minor groove binders. The substantially negative Δ*C*p^obs^ values support the general assumption that binding leads to extensive dehydration of apolar (rather than polar) groups (see Privalov et al. 2007 for a summary). The contribution from dehydration of the protein, Δ*C*p^prot^, calculated using the above (averaged) Eq. (2), is given in orange and the contribution of the DNA (Δ*C*p^DNA^ in blue) is then the difference from the observed values. The most striking feature of this data set is that whilst the protein contributions do not differ greatly for binding in the two grooves, the heat capacity changes from dehydration of the DNA are very different: substantially negative in the major groove but only slightly so for the minor groove. It is clear that Δ*C*p for the DNA and protein surfaces (per Å^2^) is not the same: i.e., the hydration characteristics of the DNA grooves differ from those of the proteins.

### Analysis of the interface

The programs Naccess and PDIviz were used to determine the accessible surface areas of the protein domains, the free DNA and their complexes using just two categories of surface: apolar (including aromatic) and polar/charged. From these data, values of ΔASA, the change (reduction) in accessible surface area on forming the complexes were calculated for both the protein and DNA components. The aromatic component was not separately assessed since aromatic rings are rarely exposed on the surface of proteins and in duplex DNA it is only the edges of the bases that are exposed, not the aromatic rings.

Table 1 lists values of ΔASA^prot^, the reduction in accessible surface area of the proteins on forming their complexes and the calculated heat capacity changes, Δ*C*p^prot^, resulting from occlusion of their surface area, using Eq. (2). Values of Δ*C*p^prot^ were then subtracted from the observed Δ*C*p^obs^ values to yield values of Δ*C*p^DNA^, the heat capacity change attributable to dehydration of the DNA on forming the complex. These values of Δ*C*p^DNA^, together with the changes in apolar and polar surface areas of the DNA (ΔASA^DNA^) for each complex were substituted into the equation:

3 $$ \Delta C{\text{p}}^{\text{DNA}} \left( { 2 5\;^\circ {\text{C}}} \right){ = [}C{\text{p}}^{\text{apolar}} \left( { 2 5\;^\circ {\text{C}}} \right) \, \times \Delta \left( {\text{ASA}} \right)^{\text{apolar}} ] { + [}C{\text{p}}^{\text{polar}} \left( { 2 5\;^\circ {\text{C}}} \right) \times \Delta \left( {\text{ASA}} \right)^{\text{polar}} ]. $$

**Table 1:**
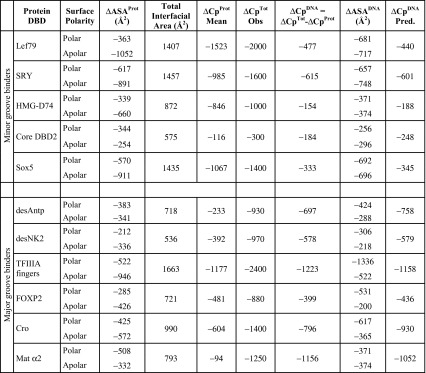
Observed heat capacity effects resulting from binding DBDs to the minor and major grooves of DNA separated into contributions from the dehydration of protein and DNA

**Fig. 2:**
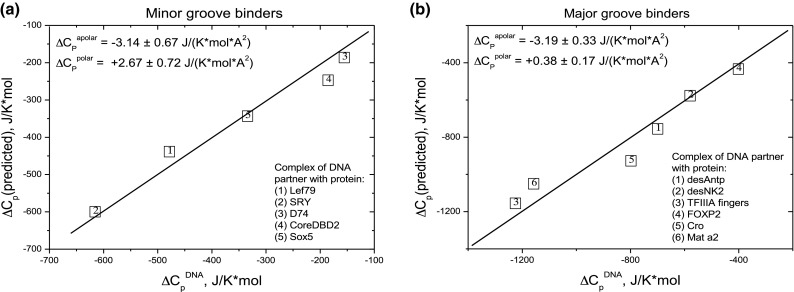
The correlation of experimental Δ*C*p^DNA^ values, *x*-axis, with those predicted Δ*C*p (predicted), *y*-axis, on the basis of calculated ΔASA_apolar_ and ΔASA_polar_ values and two equations of type (3) with the fitting parameters shown in the insets. The line functions on the graphs, [Δ*C*p(predicted) = Δ*C*p^DNA^] correspond to identity of the predicted and experimental heat capacity. The numbers in the boxes correspond to the individual protein/DNA complexes

Δ*C*p values in J K^−1^ mol^−1^. Values of predicted Δ*C*p^DNA^ were calculated using the magnitudes of ΔASA^polar^ and ΔASA^apolar^ in the penultimate column and the corresponding equations for the minor and major groove binders given in Fig. 2 and Table 2 (below)

Thermodynamic data obtained for: Lef79, SRY, Sox5, HMG-D74 (Dragan et al. 2004); Core DBD2 (Dragan et al. 2003); desAntp and desNK2 (Dragan et al. 2006); TFIIIA (Liggins and Privalov 2000); FOXP2 (Morris et al. 2018); Cro (Takeda et al. 1992); Mat α2 (Carra and Privalov 1997). Sox5/DNA complex structure—unpublished data from (Read et al. 2018)

### The minor groove

Five complexes were used, three of which are HMG boxes (see Dragan et al. 2004). D74 is a truncated form of the non-sequence specific (NSS) Drosophila HMG-D protein and includes only the minimal HMG box, i.e., excludes the highly basic 26 residue C-terminal tail included in the D100 construct. Lef79 is likewise the minimal HMG box from the mouse sequence-specific (SS) transcription factor LEF-1, similarly missing its basic 8-residue C-tail present in Lef86. Data for the longer versions of these two DBDs have not been used since their basic C-terminal tails do not bind in the minor groove but extend across the major groove and make only non-specific ionic links. SRY81 is the SS HMG box from human SRY: this includes a short C-terminal tail that tracks along the minor groove adjacent to the HMG box itself and the same is true for the HMG box from mouse Sox5. The AT-hook motif ‘Core DBD2’ is the minimal 10-residue ‘core’ element representing the second AT-hook from HMGA1, the central RGR element of which sits on the floor of the minor groove.

#### The major groove

Six complexes have been used for the major groove analysis. Homeodomains insert a recognition helix into the major groove, but additionally have N-terminal extensions into the minor groove. To restrict consideration to just the major groove, Fig. 1 gives data for the truncated forms of the Antennapedia and NK2 homeodomains that lack their N-terminal extensions: desAntp and desNK2 (Dragan et al. 2006). It also includes data for the Mat α2 homeodomain that retains a very short N-terminal extension in the minor groove (Carra and Privalov 1997). FOXP2 inserts the third α-helix of its forkhead domain into the major groove and there are very few minor groove contacts in this case (Morris et al. 2018). TFIIIA is a three zinc-finger element from the xenopus protein (Wuttke et al. 1997; Liggins and Privalov 2000). Thermodynamic data for DNA binding of the Cro repressor dimer are from Takeda et al. 1992.

In Eq. (3) the values of Δ(ASA)^apolar^ and Δ(ASA)^polar^, the apolar and polar surface areas of DNA binding sites, are independent variables. For the minor groove complexes there are five sets of apolar and polar ΔASAs variables and six sets of variables for the major groove complexes. The searched-for parameters, the coefficients *C*p^*i*^, are taken as constants and represent the surface-normalized heat capacity effects of dehydrating unit surface area of apolar and polar surface on the DNA. These were initially estimated using Eq. (3) and then the regression programme in Origin was applied to each set of ΔASA^*i*^ variables. Table 2 gives the resulting equations for each groove. To display how effectively the calculated coefficients express the experimental data, a graph was plotted for each groove of the experimentally observed heat capacities against those predicted using the derived coefficients, see Fig. 2. The diagonals, having a slope of unity, represent exact correspondence between the observed Δ*C*p^DNA^ and that calculated using the averaged values of *C*p^*i*^ given in Table 2.

**Table 2:** Δ*C*p^(25C)^ = [Δ(ASA) × *C*p^*i*^]^Apolar^ + [Δ(ASA) × *C*p^*i*^]^Polar^ is Eq. (3) used to express the predicted heat capacity, Δ*C*p, in terms of the reductions in accessible surface area, Δ(ASA), in Å^2^ and the heat capacity coefficients *C*p^*i*^ in J K^−1^ mol^−1^ [Å^2^]^−1^

Component	Cp^*i* Apolar^	Cp^*i* Polar^
Protein	− 1.79 ± 0.40	+ 0.98 ± 0.35
DNA major groove	− 3.19 ± 0.33	+ 0.38 ± 0.17
DNA minor groove	− 3.14 ± 0.67	+ 2.67 ± 0.72

## Discussion

The first comment is that in both grooves the apolar coefficients are negative, whilst both polar coefficients are positive—just as with proteins. The precise coefficients immediately show why the contribution to Δ*C*p from dehydration of the DNA is much less in the minor groove than in the major groove (see Fig. 1) despite not dissimilar interfacial contact areas: in the minor groove the apolar and polar coefficients are of comparable magnitude but of opposite sign, leading to a low net contribution, whereas in the major groove the positive polar coefficient is small in magnitude relative to the apolar so that the apolar dominates, resulting in strongly negative net heat capacity changes.

The essence of the equations derived for the two grooves can be summarized as follows: the heat capacity effect of forming an apolar interface is very similar in the two grooves and is characterized by a negative value of Δ*C*p^apolar^ = − 3.2 J K^−1^ mol^−1^ [Å^2^]^−1^, a situation similar to the formation of an apolar interface in proteins—for which the average Δ*C*p^apolar^ is − 1.79 J K^−1^ mol^−1^ [Å^2^]^−1^ (Eq. 2). The uniformity of Δ*C*p^apolar^ for the two grooves of DNA must result from the fact that the walls of both grooves consist of exposed sugar rings. Furthermore, the quantitative difference between apolar DNA surface and that of the averaged apolar (largely aliphatic) surface of polypeptide chains reflects differences in their effects on the water bound to them.

In contrast, the formation of polar interfaces is very different in the two grooves. In the minor groove, a large and positive value of Δ*C*p^polar^ = + 2.67 J K^−1^ mol^−1^ [Å^2^]^−1^ is observed, substantially larger than the values found for the formation of a polar interface in proteins, that average to Δ*C*p^Polar^ = + 0.98 J K^−1^ mol^−1^ [Å^2^]^−1^ (Eq. 2). This unusually large positive polar coefficient characteristic of the minor groove can only be a consequence of displacing the ordered ice-like water known to be characteristic of this groove (Kopka et al. 1983: Chiu et al. 1999) the ordering of which is based on the regularity of N3 of A and O2 of T groups in AT-rich regions and is, therefore, a property of the polar surface of the minor groove. In this context, it is worth recalling that the heat capacity of liquid water (4.18 J g^−1^ K^−1^ at 25 °C) is about twice that of ice (2.03 J g^−1^ K^−1^ at − 10 °C). Release of this water gives rise to unusually positive enthalpies and entropies, a notable characteristic of protein binding to the minor groove (Privalov et al. 1999, 2007 Dragan et al. 2004; Privalov and Crane-Robinson 2017, 2018).

**Fig. 3:**
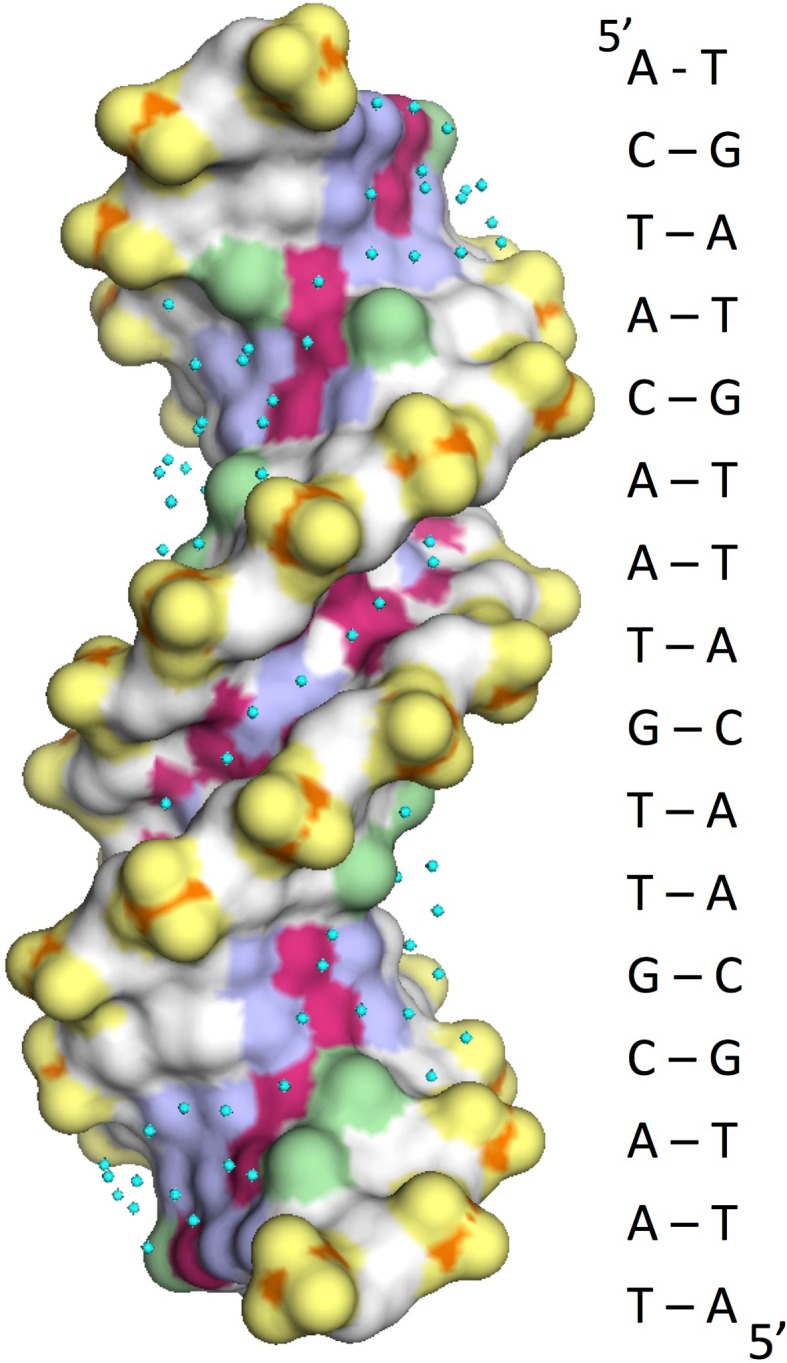
Representation of a 16 bp duplex solved at 1.6 Å resolution (Narayana and Weiss 2009) showing the apolar and polar surface characteristics. Apolar: carbons in white and the methyl groups of T in green. These constitute the walls of both grooves. Polar: red (negative) and blue (positive). These make up the bottom of both grooves. Phosphate groups are separately designated in yellow and water molecules are shown as cyan dots. Note the very regular array of eight waters covering six bp in the central minor groove (five ATs interrupted by a single GC). Water molecules in the major groove are more haphazardly positioned

In the major groove a Δ*C*p^polar^ of only + 0.38 J K^−1^ mol^−1^ [Å^2^]^−1^ implies that the water bound to the irregularly spaced polar H-bond donor and acceptor groups that run along the base of the major groove has a structure that differs little from that of bulk water. Figure 3 shows a crystallographic structure of a 16 bp duplex having high enough resolution (1.6 Å) to define a substantial number of the hydrating water molecules: distinction between the ordered array in the minor groove and the relatively disordered hydration of the major groove is very apparent. A substantial difference between the grooves as regards their hydration is supported by assessments of the effective dielectric constant within the two grooves: whereas major groove water is not too different from the bulk, exhibiting an apparent dielectric constant of  ~ 50 D, water in the AT-rich minor groove appears to have a dielectric constant of only ~ 20 D (Barawkar and Ganesh 1995; Jin and Breslauer 1988). Although there is no direct relationship between the dielectric constant and the thermodynamic parameters of the hydrating water, these data demonstrate a substantial difference in the state of the water in the two grooves.

Protein/DNA complexes normally include multiple salt links between phosphates and lysine/arginine sidechains, the formation of which must be accompanied by dehydration events, so the question arises as to whether these ionic bonds are relevant to the Δ*C*p measurements made here? Experiments on a variety of DBD/DNA complexes (both sequence-specific and non-sequence specific) have shown that the enthalpy of the binding interaction is independent of the salt concentration—despite large changes in the affinities—i.e., the salt links are non-enthalpic. This means they make no contribution to the measured Δ*C*p (see Dragan et al. 2004; Takeda et al. 1992; Ladbury et al. 1994). Although this conclusion was questioned in the case of IHF binding to DNA (Holbrook et al. 2001), IHF represents an example of salt-dependent protein refolding on binding to DNA (see Swinger and Rice 2004)—with consequent enthalpic changes—rather than a direct enthalpic effect of forming/breaking ionic links with the DNA. The dehydration processes reported on by the Δ*C*p measurements, therefore, take place within the grooves, not along the phosphodiester backbone.

An attempt previously made to establish a relationship between observed heat capacity changes on complex formation and apolar/polar coefficients (Uedaira et al. 2003) concluded that Δ*C*p^apolar^ ~ − 4.7 J K^−1^ mol^−1^ [Å^2^]^−1^ and Δ*C*p^polar^ ~ + 2.5 J K^−1^ mol^−1^ [Å^2^]^−1^ for a group of ten DNA/DBD complexes, overall values derived without separating the protein and DNA contributions. Although of the same sign as found in the present analysis, these values average the protein and DNA contributions and are of larger magnitude than reported here. The differences are probably also a consequence of using experimental heat capacities uncorrected for refolding (that results in excessively negative values of Δ*C*p^obs^)—as well as not making any distinction between the grooves.

The parameters derived here for dehydration of the DNA grooves raise the question as to the physical basis of the sign and magnitude of observed Δ*C*p values. The simplest view of a negative Δ*C*p for the dehydration of apolar surface is that water molecules hydrating hydrophobic groups in free solution are oriented by the asymmetric attraction from the bulk water and in consequence are less restricted and constrained by hydrogen bonding to each other than are waters in the bulk liquid. This results in such oriented waters having an enhanced heat capacity, so that when shed into the bulk solution, their heat capacity drops. In physical terms such an explanation implies that the water hydrating polar groups in free solution is very tightly H-bonded, i.e., more constrained than the molecules of bulk water: its loss on complex formation, therefore, results in an increase in its heat capacity (Bergqvist et al. 2004).

## Summary

Treating a change in the heat capacity on forming a DNA–DBD complex as a proxy for the change in hydration of the surfaces in question, this data analysis leads to the conclusion that the water hydrating apolar DNA surface is essentially the same in both grooves (when expressed on a per Å^2^ basis), presumably as a consequence of similar exposure of sugar rings on the walls of both grooves. In contrast, the hydration of polar surface is very different in the two grooves: in the minor groove the unusually large heat capacity increase must be a consequence of the release of the highly ordered (‘ice-like’) water bound to the polar atoms on the edge of the bases. In the major groove, the water hydrating polar groups does not differ greatly from bulk water because these polar groups do not have the regularity and spacing appropriate for the formation of ordered water structures, unlike in the minor groove.
